# Workplace Bullying and Occupational Stress Among University Teachers: Mediating and Moderating Factors

**DOI:** 10.5964/ejop.v15i2.1611

**Published:** 2019-06-07

**Authors:** Naima Akhtar Malik, Kaj Björkqvist

**Affiliations:** a Department of Social Sciences, Åbo Akademi University, Vasa, Finland; Webster University Geneva, Geneva, Switzerland; Aristotle University of Thessaloniki, Thessaloniki, Greece

**Keywords:** workplace bullying, occupational stress, university teachers, mediator, moderator

## Abstract

In the study, it is explored whether exposure to workplace bullying predicts symptoms of occupational stress, and whether this association is mediated by interpersonal relationships, and moderated by sex and nationality. A sample of 610 university teachers from Pakistan (196 males, 133 females) and Finland (152 males, 129 females) completed an online questionnaire. A conditional process model was applied using the PROCESS programme. Workplace bullying served as predictor, stress symptoms as predicted variable, relationships with (a) colleagues and (b) family as mediators, and sex and country as moderators. As expected, workplace bullying had a significant effect on stress symptoms, which was mediated by family relationships but not by relationships with colleagues. Neither sex nor country had a moderating effect. Positive family relationships thus mediate the stressful impact of workplace bullying, and this was the case for both sexes and both nationalities.

Workplace bullying and occupational stress are both well-researched phenomena within organizational psychology (Einarsen, Hoel, Zapf, & Cooper, 2003). Workplace bullying has been suggested to be one of the most stressful phenomena that may occur (Duffy & Brown, 2018; Hauge, Skogstad, & Einarsen, 2010; Pheko, 2018), and it has injurious effects on the victim’s physical and psychological health (Duffy & Sperry, 2012; Giorgi, Arcangeli, Mucci, & Cupelli, 2015; Giorgi et al., 2016; Sojo, Wood, & Genat, 2016).

Workplace bullying is a serious issue also in educational institutions (Shelley et al., 2017), and there is an urgent need to manage the problem (Hauge, Skogstad, & Einarsen, 2010). It is an ethical and moral obligation of managers and supervisors to protect the employees from an unfriendly environment (Pheko, 2018).

When an individual becomes a victim of workplace bullying, his or her life quality changes; the victim’s emotional state and relationships also change. It affects negatively the work capacity of individuals, groups, and organizations (Membere et al., 2015). Bullying does not only harm friendships within the occupational social network, it also causes a state of general frustration which indirectly may affect family relationships negatively (Duffy, 2018). Lewis and Orford (2005) found that workplace bullying has a negative impact over time on close relationships; the stress caused by workplace bullying interacts with other stressful circumstances in the individuals’ life, putting strain also on family relationships.

The relationships between bullying, well-being, and health have been investigated (Einarsen & Nielsen, 2015; Park & Ono, 2017; Yragui, Demsky, Hammer, Van Dyck, & Neradilek, 2017) but mediating variables between workplace bullying and occupational stress are relatively little studied. It has been shown that one of the major sources of occupational stress is interpersonal conflict at work (Hahn, 2000; Hammen, 2006; Narayanan, Menon, & Spector, 1999a; Spector & Jex, 1998; Stoetzer et al., 2009); accordingly, relationships with colleagues could be a mediating variable. A victim from workplace bullying could still have good relationships with some colleagues, and this fact could alleviate the negative symptoms of being bullied by others. Likewise, good relationships with one’s own family may be a factor mitigating negative effects of workplace bullying. For instance, Repetti and Wang (2017) found that symptoms of occupational stress were affected by the level of support from one’s family. The more support, the less symptoms, and vice versa. Therefore, family relationships could well be another moderating factor.

The present study investigates whether (a) relationships with colleagues, and (b) family relationships serve as mediating variables between workplace bullying and occupational stress, by the use of conditional process modeling (Hayes, 2012; Preacher & Hayes, 2004). Sex differences in this respect are also studied, by keeping sex as a moderator in the model. Furthermore, two aggregated samples of university teachers were used, one from Finland, the other Pakistan; therefore, country belonging served as another moderator, besides sex.

There are good reasons to choose sex and country as moderators, since both have visible (physical) and invisible (value) components, and both influence group interrelation (Ayman & Korabik, 2010). Eby et al. (2005) concluded that sex differences are deep-rooted in work–family relations, and it is imperative to consider sex differences and sex role issues to completely understand the work–family interface. The inherent idea in numerous discussions on work–family disputes is that the handling of the work–family interface is much more challenging for women than men (Shockley, Shen, DeNunzio, Arvan, & Knudsen, 2017). Sex differences in workplace bullying have not been considered much, or at least, not enough (Escartín, Salin, & Rodríguez-Carballeira, 2011; Hoel, Cooper, & Faragher, 2001; Lee, 2002), However, there is evidence supporting differences in effects, forms, and frequency of workplace bullying among men and women (Rayner & Cooper, 1997). Women have been found to report workplace bullying more often than men (Björkqvist, Österman, & Hjelt-Bäck, 1994), and they report psychological effects more often than men (Niedl, 1996).

Work and family interactions are rooted in the cultural, organizational, and economic situation in which people live (Ollier-Malaterre & Foucreault, 2017). In countries where disparities among males and females are minimized, experiences within both family and work environments may be similar for both sexes, leading to lesser sex differences in work-family interactions (Casper, Harris, Taylor-Bianco, & Wayne, 2011; Fahlén, 2014). Finland is a leader among world countries in gender equality (Ministry of Social Affairs and Health, 2018). On the other hand, Pakistan faces serious challenges regarding gender equality. Malik, Björkqvist, and Österman (2017a, 2017b) found substantial differences between Finland and Pakistan regarding university employees experiences of occupational stress and burnout. Therefore, it was decided to keep country as a moderator in the present study.

The study is part of a larger project, investigating factors related to occupational stress in Pakistan and Finland (Malik et al., 2017a, 2017b). The purpose of comparing these two countries is the earnest need of developing countries to enhance their institutional practices; this can only be made possible by learning from other contexts. As developing countries are going through a rapid transition in higher education (Chaudhry, 2012), it is due time to become aware of the useful practices of developed countries. Comparisons facilitate the identification of strengths and weaknesses of educational systems, thereby suggesting ways to improve weak areas (Zhao et al., 2008).

Therefore, Pakistan, a developing country, was selected, as working conditions in the country are becoming more challenging, and work hours have increased; statistics indicate that in the year 1971-1972, 20.4% of staff used to work more than fifty-six hours a week, whereas, in 2004 the figure was 30.8% (Nadeem & Abbas, 2009). Due to this over-commitment, both male and female employees are facing problems with fulfilling family responsibilities, which leads into a conflict between work and family life (Nadeem & Abbas, 2009). Finland, a developed country, was chosen as Finland is believed to have excellent work environments compared to most other countries. As stated by different life indexes, Finland does better in various measures of well-being in comparison to most of the world. It is placed amongst the best in personal safety, education, and work-life balance (OECD, 2015). Finland does well above average in environmental quality, personal safety, civic engagement, social connections, personal well-being, housing, and work-life balance (OECD, 2015).

## Occupational Stress

A sense of psychological pressure due to experiencing different stressors at work is known as occupational stress (Karasek & Theorell, 1990). Occupational stress has been claimed by some to be an unavoidable phenomenon of contemporary age (Stojanović, Milenović, & Marković, 2012). Research has shown that teaching is a stressful profession, and that occupational stress can influence job satisfaction and motivation of teachers (Maphalala, 2014). It may also enhance negative job attitudes (Aquino & Thau, 2009; Barling, Kelloway, & Frone, 2005). Chronic stress may further lead to behavioral and cognition problems (Cohen, Janicki-Deverts, & Miller, 2007).

Emotionally and physically demanding work with little control over one’s work situation is especially stressful (Grosch & Sauter, 2005). Some amount of work stress is unavoidable in every occupation, but experiencing stress for an extended period can be very damaging for one’s health, as it can be a cause for aggression, job dissatisfaction, burnout, truancy, anxiety, fatigue, substance abuse, and poor performance (Bodenmann, Meuwly, Bradbury, Gmelch, & Ledermann, 2010; Cropanzano, Rupp, & Byrne, 2003; Johnson, Perry, & Rosensky, 2002; Martinussen, Richardsen, & Burke, 2007; Morash et al., 2008; Podsakoff, LePine, & LePine, 2007; Schwabe & Wolf, 2010; Thoits, 2010; Violanti et al., 2016; Wang, 2005).

There are several contextual factors which have been recognized as facilitators of occupational stress, such as poor organizational support, job insecurity (Abbasi, Araban, & Aalipour, 2018; Malik et al., 2017a, 2017b; Milner, Witt, LaMontagne, & Niedhammer, 2018), time pressure (Prem, Ohly, Kubiceki, & Korunka, 2017; Prem, Paškvan, Kubicek, & Korunka, 2018), job ambiguity, job conflict, workplace bullying (Attell, Brown, & Treiber, 2017), excessive work load (Buchanan, 2010; Smithers & Robinson, 2003), and job demand and lack of control (Fila, Purl, & Griffeth, 2017). Another emerging job stressor which has caught the attention of researchers is the economic crisis which emerged in 2008 and had a negative effect on working conditions and the mental and general health of employees (Mucci, Giorgi, Roncaioli, Perez, & Arcangeli, 2016). It may have had an increasing effect on the prevalence of workplace bullying (D’Cruz, Noronha, & Beale, 2014). Studies have corroborated the fact that economic crises lead to an increase in unemployment, heavy workload, staff reduction, and decrease in wages, and that they are related to increased levels of mood disorders, anxiety, depression, dysthymia, and suicide (Mucci et al., 2016).

## Workplace Bullying

Workplace bullying has been defined as repeated activities with the aim of bringing mental (but sometimes also physical) pain to the victim(s), and directed towards one or more individuals who, for one reason or another, are not able to defend themselves (Björkqvist et al., 1994). Workplace bullying has also been referred to as work harassment (Brodsky, 1976) and mobbing at the workplace (Leymann, 1992). A typical feature which distinguishes bullying from general aggression is the imbalance of power between perpetrator and victim (Einarsen et al., 2003). Another typical feature is the repetitiveness of the aggressive behaviour, which little by little breaks down the victim.

The percentage of employees who may be considered as victims of workplace bullying vary from workplace to workplace and study to study, depending partly on what criteria have been used. Workplace aggression, in its various forms, is extremely common, and has been experienced by 96% of employees; that is, by almost everybody (Porath & Pearson, 2010). The point prevalence of victimisation of workplace bullying is usually around 10%. Nielsen et al. (2009) found that 6.8% in a sample of 2539 Norwegian employees could be defined as victims. In another Norwegian study (Einarsen & Skogstad, 1996), the prevalence was 8.6%. The Norwegian figures are relatively low in comparison with findings from other European countries: e.g., UK,10.6% (Hoel et al., 2001); Belgium 3-20% (Notelaers, De Witte, Vermunt, & Einarsen, 2006); and Lithuania 23% (Malinauskiene, Obelenis, & Dopagiene, 2005).

Björkqvist et al. (1994) found that 11% of teachers at a university in Finland had been exposed to workplace bullying during the last six months. They also found that victims scored significantly higher than others on depression, anxiety, and aggression. Stress symptoms, depression, and psychosomatic symptoms are common among victims of workplace bullying (Bernotaite & Malinauskiene, 2017; Matthiesen & Einarsen, 2004; Nielsen, Matthiesen, & Einarsen, 2008). Some victims show signs of PTSD (Björkqvist et al., 1994; Matthiesen & Einarsen, 2004; Mikkelsen & Einarsen, 2002; Leymann, 1992). In especially severe cases, suicides are known to have taken place (Leymann, 1992).

A meta-analysis (Nixon et al., 2011) found that workplace bullying resulted in numerous harmful health problems like insomnia, back- and headaches, exhaustion, alcohol use, and stomach problems. Bullying is also a cause of frustration and depression (Yıldırım, 2009), which evoke interpersonal conflict (Reknes, Einarsen, Knardahl, & Lau, 2014), and interpersonal conflict unleash deviant behaviour (Spector et al., 2006). Employees, who experience themselves to be victims of workplace bullying, report increased levels of stress, anxiety, and depression, which is injurious for their personality (Fox & Stallworth, 2005)

## Relationships With Colleagues

Interpersonal relations at work play a crucial role in ensuring an environment of trust and positive emotions. Good interpersonal relationships are not enough to improve employees’ performance, but they may contribute significantly to it. As studies have shown, employees’ work ability, job satisfaction, and career success are directly related to the quality of workplace relationships (Markiewicz, Devine, & Kausilas, 2000; Morrison, 2004; Sias & Cahill, 1998).

Healthy workplace relationships are considered a key factor in reducing occupational stress. Interpersonal relationships strongly influence the well-being and health of an individual. Appelberg, Romanov, Heikkilä, Honkasalo, and Koskenvuo (1996) conducted a six year follow up study among 15,000 employees and found that poor interpersonal relationships affected work ability negatively.

Bruk-Lee and Spector (2006) found that interpersonal conflicts at work is one of the major causes of stress. It is the most referred to source of stress for college professors (Narayanan, Menon, & Spector, 1999b). Liu (2004) found that interpersonal conflict is a key source of stress among faculty and support staff in both a Chinese and an American university sample.

Keenan and Newton (1985) reported that 74% of stressful incidents occurred due to social interaction with bosses, assistants, or colleagues. Employees from different occupations mention interpersonal matters as the utmost upsetting stressor at work (Smith, 1995).

The level of burnout can also be predicted by the nature of interpersonal relationships among teachers (Cano-García, Padilla-Munoz, & Carrasco-Ortiz, 2005; Dorman, 2003; Friedman, 2003; Gavish & Friedman, 2010; Grayson & Alvarez, 2008; Leung & Lee, 2006; Skaalvik & Skaalvik, 2009, 2011; Van Droogenbroeck, Spruyt, & Vanroelen, 2014). A meta-analysis by Spector and Jex (1998) showed that there is a positive association between interpersonal conflict and negative emotional states, such as depression, anxiety, and frustration.

## Family Relationships

The association between family relationships and occupational stress is much less researched than the association between relationships with colleagues and occupational stress. However, job and family are closely interrelated and interdependent, and incidents in one area affects the quality of life in the other area (Allen, Herst, Bruck, & Sutton, 2000; Sarantakos, 1996). Demsky, Ellis, and Fritz (2014) found that there is a relationship between workplace aggression and family conflict; when there is an increase in workplace aggression, family conflicts also tend to increase. Job stressors affect family life and vice versa (Antoniou, Davidson, & Cooper, 2003).

Repetti and Wang (2017) found that everyday job stressors affect family relationships in positive and negative ways depending upon the provision of support from family members. When a person experience stressors and stress at work, it usually provokes anger, irritation, and frustration at home, too (Narayanan et al., 1999a; Nixon et al., 2011).

However, all studies have not found an association between family relationships and occupational stress. For instance, Wilczyński et al. (2015) found that family had no impact on job involvement and burnout.

## Aim and Hypotheses of the Study

The present study aims at investigating whether workplace bullying has an effect on (is a predictor of) occupational stress symptoms, and whether this effect is mediated by relationships with colleagues, and/or family relationships, and moderated by sex (male vs. female respondents) and country belonging (Pakistan vs. Finland).

The following hypotheses were set up for a conditional process model analysis:

H1: Relationships with colleagues are expected to be a significant mediator between workplace bullying and occupational stress.

H2: Family relationships are expected to be a significant mediator between workplace bullying and occupational stress.

H3: Sex is expected to have a moderating effect in the process, the effect being stronger for males than for females.

H4: Country is expected to have a moderating effect in the process, the effect being stronger in Pakistan than in Finland.

## Method

### Sample

The sample consisted of university teachers from 28 conveniently sampled public universities in Pakistan and Finland. For the collection of data, the official e-mail addresses of university teachers employed at these universities were obtained from the university websites. An e-mail was sent out to each address, with a link to an online survey which could be filled in only once. A total of 610 responses were received. It is not possible to estimate an exact response rate as it is impossible to verify the number of valid and active e-mail addresses. Some of the e-mails bounced back to the sender.

The distribution of female and male teachers in Pakistan and Finland is presented in Table 1. The mean age was 42.1 years (*SD* = 10.1) for females, and 42.3 years (*SD =* 10.0) for males; the age difference between the sexes was not significant. The mean age was 37.2 years (*SD* = 7.6) for the Pakistani teachers and 48 years (*SD* = 9.4) for the Finnish teachers.

**Table 1:** Number of Female and Male University Teachers in Pakistan and Finland Taking Part in the Study

Country	Females		Males		Total	
	*n*	%	*n*	%	*n*	%
Pakistan	133	22	196	32	329	54
Finland	129	21	152	25	281	46
Total	262	43	348	57	610	100

### Instrument and Analysis

The study included four scales: (1) *workplace bullying* was measured with subscales from DIAS-Adult (Österman & Björkqvist, 2009); (2) *occupational stress symptoms* were assessed with the Work Stress Symptoms Scale (Björkqvist & Österman, 1992); (3) *relationships with colleagues* were measured with an instrument designed specifically for this study. Some of the items were adapted from the Relationship Structures (ECR-RS) Questionnaire by Fraley, Heffernan, Vicary, and Brumbaugh (2011). (4) *Family relationships* were measured with an instrument developed particularly for this study by the first author; items from (Jackson & Maslach, 1982) were also adapted for this scale.

Sample items of the four scales, as well as their reliability scores (Cronbach’s α), are presented in Table 2. The responses alternatives for all scales were on a five-point scale ranging from 0 = never to 4 = very often, or from 0 = strongly disagree to 4 = strongly agree.

**Table 2:** Sample Items and Cronbach’s Alphas of the Scales in the Study (N = 610)

*Workplace Bullying* (28 items, α = .97)
Sample items: Has someone at your workplace …? Yelled at you; Quarrelled with you; Interrupted you bluntly when you were talking; Been ironic towards you; Ridiculed you in your absence; Spoken badly about you to someone else; Made false accusations about you; Refused to talk to you; Refused to look at you.
*Occupational Stress Symptoms* (12 items, α = .94)
Sample items: Do you feel, as a consequence of your work …? Exhaustion; Difficulties to concentrate; Weariness and feebleness; Insomnia (disturbed sleep); Nervousness, Irritation; Tension; Depression; Indifference towards everything.
*Relationships with Colleagues* (22 items, α = .95)
Sample items: I have good working relations with most of my colleagues; My colleagues show fairness in their interactions; I trust my colleagues; Me and my colleagues work well together; I think that I can turn to my colleagues in time of need; I feel comfortable opening up to my colleagues.
*Family Relationships* (18 items, α = .90)
Sample items: I spend enough time with my family members; I often find time to go for an outing with my family; I usually get upset at family gatherings due to tiredness^a^; I spend off-hours away from family^a^; I am involved with family matters; I find enough energy after work to attend family gatherings.

^a^Reversed scoring.

To avoid a possible common method bias (CMB), great care was taken to examine the items to avoid vague, unclear and unfamiliar terms, as suggested by researchers (Lindell & Whitney, 2001; Podsakoff, MacKenzie, Lee, & Podsakoff, 2003). Anonymity and confidentiality was emphasized so that the participants could respond as honestly as possible (Chang, van Witteloostuijn, & Eden, 2010). After collecting the data, the Harman’s single-factor test was conducted which showed that a one-factor solution accounted for only 29.3% of the variance, well below the critical threshold of 50%, indicating that CMB was not a matter of concern.

The mediation analysis was conducted with the programme PROCESS (Hayes, 2013), based on bootstrapping. Bootstrapping builds an empirical approximation of the sampling distribution and uses this to construct confidence intervals for the indirect effects. In this particular case, a 5,000 bootstrap sample was applied with the confidence interval set at 95%. Bootstrapping allows multiple mediators and moderators in the same model. The researcher may not only study the collective indirect effect of various mediators, but also compare the intensity of indirect effects and the range to which a mediation process is conditional on a moderator, i.e. a moderated mediation (Hayes, 2012; Preacher & Hayes, 2004).

For the measurement of the effect size of the mediation paths, the c’/c ratio was used. Preacher and Kelley (2011) suggested the use of k^2^ (kappa-squared) as a measure of effect size; however, Wen and Fan (2015) showed that Preacher’s and Kelley’s calculations were mathematically incorrect, and k^2^ should not be used. Instead, they suggested the use of the ratio between the indirect and the direct effect as a measure for the effect size of mediation paths. Accordingly, it is used here. The ratio between the indirect and the total effect is also reported.

### Ethical Considerations

Participation was completely voluntary, participants were all adults, and full confidentiality was guaranteed. The study adheres to the principles concerning human research ethics of the Declaration of Helsinki (World Medical Association, 2013), as well as guidelines for the responsible conduct of research of The Finnish Advisory Board on Research Integrity (2012).

## Results

### Correlations Between the Variables of the Study

Pearson’s correlation coefficients between the variables of the study are presented separately for male and female teachers (Table 3), and for Pakistan and Finland (Table 4). All correlations were highly significant, for males, for females, and for both countries.

**Table 3:** Pearson’s Correlations Between the Variables of the Study for Male and Female Teachers (N = 610)

Variable (Scale)	1	2	3	4
1. Stress Symptoms	−	-.47***	-.67***	.59***
2. Colleague Relationships	-.45***	−	.50***	-.51***
3. Family Relationships	-.67***	.45***	−	-.45***
4. Workplace Bullying	.44***	-.54***	-.40***	−

*Note.* Correlations for male teachers are presented above the diagonal, and correlations for female teachers are presented below the diagonal.

***p ≤ .001.

**Table 4:** Pearson’s Correlations Between the Variables of the Study for Teachers From Pakistan and From Finland (N = 610)

Variable (Scale)	1	2	3	4
1. Stress Symptoms	−	-.43***	-.65***	.56***
2. Colleague Relationships	-.52***	−	.45***	-.51***
3. Family Relationships	-.73***	.48***	−	-.42***
4. Workplace Bullying	.45***	-.51***	-.44***	−

*Note.* Correlations for teachers from Pakistan are presented above the diagonal, and correlations for teachers from Finland are presented below the diagonal.

***p ≤ .001.

### Conditional Process Analysis

**Figure 1:**
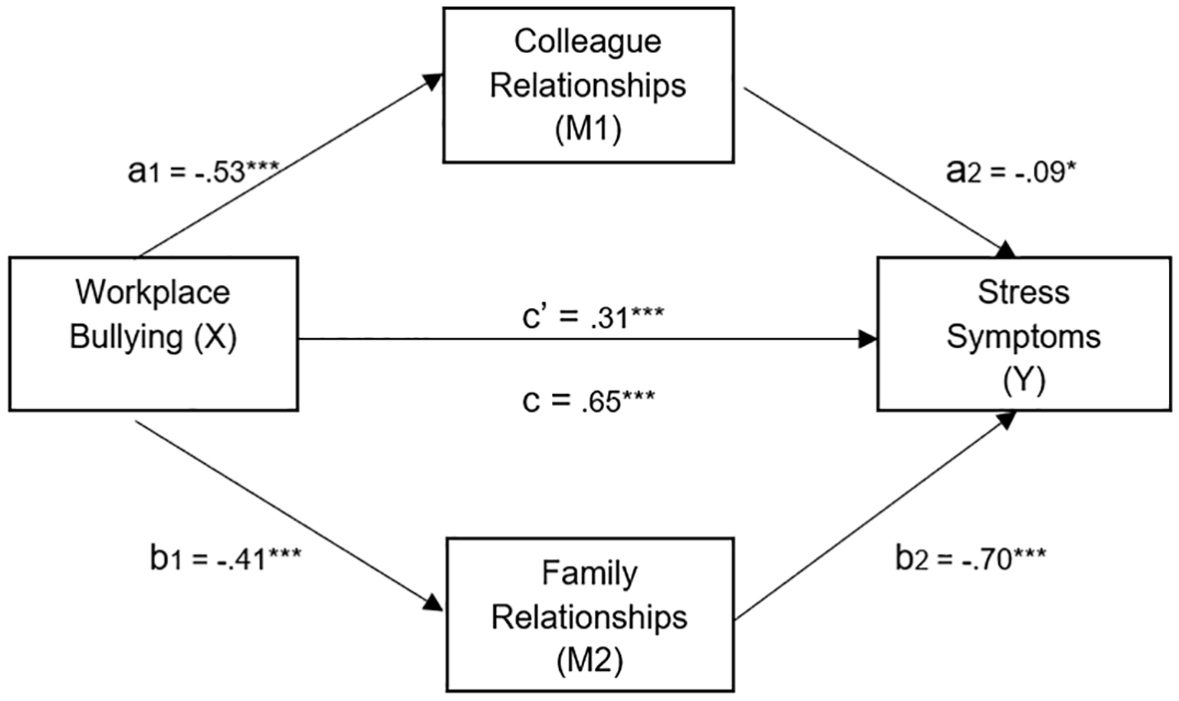
Results of a conditional process analysis of the effect of Workplace Bullying (X) on symptoms of Occupational Stress (Y), with Relationships with Colleagues (M1) and Family Relationships (M2) as mediators. *Note.* See also Table 5. *p ≤ .05. ***p ≤ .001.

A conditional process model was applied with workplace bullying as the predictor, stress symptoms as the outcome variable, relationships with colleagues and family relationships as mediators, and sex and country as moderators. PROCESS (Hayes, 2013) was used, to test the mediation and moderation hypotheses. In the present scenario of investigating the effect of workplace bullying on stress symptoms, the mediating roles of relationships with colleagues and family respectively, with sex and country as moderators, were analysed. The results are presented in Tables 5 – 6 and Figure 1.

**Table 5:** Mediating Effects of Relationships With Colleagues (M1) and Family Relationships (M2) Between Workplace Bullying (X) and Occupational Stress (Y)

Mediating Effect	*B*	*p*	95% CI	
			*LL*	*UL*
Mediating Effect of Colleague Relationships				
(a1) X‒M1	-.53	≤ .001	-.60	-.46
(a2) M1‒Y	-.09	.031	-.17	-.01
(a1 + a2) X‒M1‒Y	.04		-.01	.09
Ratio of indirect to direct effect: .15				
Ratio of indirect to total effect: .07				
Mediating Effect of Family Relationships				
(b1) X‒M2	-.41	≤ .001	-.47	-.34
(b2) M2‒Y	-.70	≤ .001	-.79	-.62
(b1 + b2) X‒M2‒Y	.29		.22	.36
Ratio of indirect to direct effect: .91				
Ratio of indirect to total effect: .44				
Model Summary				
Total effect (c)	.65	≤ .001	.56	.73
Direct effect (c’)	.31	≤ .001	.23	.39
Indirect effect (ab)	.33		-.33	-.15
Ratio of indirect to direct effect: 1.06				
Ratio of indirect to total effect: .65				

*Note.* CI = confidence interval; *LL* = lower limit; *UL* = upper limit. *N* = 610. See also Figure 1.

Table 5 presents the results of the mediation analyses. As can be seen, Relationships with Colleagues (M1) did not mediate the path between Workplace Bullying (X) and Occupational Stress, since zero was included in the confidence interval. H1 did not receive support.

However, Family Relationships (M2) did serve as a mediator, and the effect size as measured with the ratio between the indirect and the direct effect was high, .91. H2 did receive support. A graphic presentation of the results is presented in Figure 1.

Since Relationships with Colleagues (M1) did not serve as a mediator as had been hypothesised, it was pointless to further investigate whether Country and Sex served as moderators of the pathway over M1. Instead, it was analysed whether they moderated the pathway over M2, Family Relationships. The results are presented in Table 6.

**Table 6:** Moderating Effects of Country and Sex on the Mediating Effect of Family Relationships (M2) on the Relationship Between Workplace Bullying (X) and Occupational Stress (Y) (N = 610)

Interaction Effect	*B*	*p*	95% CI	
			*LL*	*UL*
Interaction Effect of Country x Family Relationships				
(b1) X‒M2	-.22	.002	-.41	-.09
(b2) M2‒Y	-.14	.094	-.29	.02
Interaction Effect of Sex x Family Relationships				
(b1) X‒M2	.01	.880	-.13	.15
(b2) M2‒Y	-.10	.150	-.23	.04

*Note.* CI = confidence interval; *LL* = lower limit; *UL* = upper limit.

As Table 6 shows, the confidence intervals for both potential moderators (Country and Sex) included zero. That is, none of them served as a moderator for the pathway over M2. Both H3 and H4 had to be rejected.

### Conclusions

#### Hypothesis Testing

Only one of the four hypotheses was supported by the results. The findings indicated that in this sample, family relationships (H2), but not relationships with colleagues (H1), mediated the effect of workplace bullying on occupational stress. Thus. H2, but not H1, was corroborated. H3 and H4 did not receive support. Neither country nor sex moderated the mediating effect of family relationships.

It should be noted that the results suggest a partial, but not a full, mediation for Family Relationships. In reality, it is impossible ever to unequivocally prove full mediation, since that would require that one has measured, without error, all possible mediators and suppressors (Rucker, Preacher, Tormala, & Petty, 2011).

#### Strengths and Limitations

A strength of this study is the use of mediation and moderation analysis to better explain the effect of workplace bullying on symptoms of occupational stress among university teachers. To our knowledge, no previous study has attempted to use conditional process modelling in order to shed light on the mediating effect of interpersonal relationships on the link between workplace bullying and occupational stress symptoms.

However, the present study has several limitations that should be noted. The first one concerns the representativeness of the sample. Although an attempt was made to collect data from different regions of both countries, it would be incorrect to consider the sample as fully representative. Furthermore, it is not possible to estimate an exact response rate, since it is difficult to assess the exact number of valid e-mail addresses to which the electronic questionnaire was sent.

The second limitation is that it was a cross-sectional study, which limits conclusions about causality within the conditional process model. In order to test causation, a longitudinal study is required. Reverse causation remains a possibility, and the role of third variables is not automatically ruled out by cross-sectional designs.

Third, it may be claimed that a paper-and-pencil questionnaire could have produced different results than an electronic one. However, Boyer, Olson, Calantone, and Jackson (2002) found that e-surveys are as good as manual surveys: both methods have similar response rates and receive more or less similar results.

Fourth, this sample consisted of university teachers, and results may not necessarily be generalised to other work forces. Generalisations to other countries must also be made with caution, until replications in other countries have been made.

#### Final Remarks

The central finding of this study was that family relationships (but not relationships with colleagues) had a mediating impact on the effect of workplace bullying on stress symptoms. The findings underscore the importance of having good relations with one’s family. It is noteworthy that neither sex nor country moderated this effect. This fact suggests that the mediating effect of family relationships is relatively robust, since it was found in two quite different cultures, and within both sexes, in both countries. A suggestion for future research is to investigate whether this result can be replicated in other countries around the globe. Another suggestion is to probe deeper into how this alleviating effect occurs.
